# Short-Term Surgical Outcomes and Postoperative Safety in Octogenarian Patients Undergoing Colorectal Cancer Resection: A Comparative Cohort Study

**DOI:** 10.3390/cancers18213460

**Published:** 2026-10-27

**Authors:** Albert Garcia-Nalda, Victor Admella-Clanchet, Anna Serracant-Barrera, Mariana Caraballo-Angeli, Laura Lazaro-Garcia, Claudia Codina-Espitia, Anna Nonell-Amill, Laura Mora-Lopez

**Affiliations:** Colorectal Unit, General and Digestive Surgery, Hospital Universitari Parc Taulí, 08208 Sabadell, Spainlmora@tauli.com (L.M.-L.)

**Keywords:** colorectal cancer, octogenarians, geriatric oncology, robotic surgery, postoperative complications, surgical safety, frailty, IF-VIG, prehabilitation, ERAS

## Abstract

**Background**: Surgical management of colorectal cancer (CRC) in elderly adults presents complex clinical challenges due to baseline multimorbidity and frailty. This study aimed to evaluate short-term surgical safety and postoperative outcomes in carefully selected octogenarian patients (≥80 years) compared with a younger cohort (<80 years) in a specialized colorectal unit following comprehensive geriatric evaluation, prehabilitation, and standardized minimally invasive surgery. **Methods**: A retrospective observational cohort study was conducted on 742 consecutive patients undergoing elective curative-intent resection for CRC between 2021 and 2025. Patients were stratified into two cohorts: <80 years (*n* = 604) and ≥80 years (n = 138). Clinical characteristics, frailty burden evaluated using the clinician-reported IF-VIG index, surgical techniques, histopathological staging, and 30-day postoperative morbidity were evaluated. Multivariable logistic regression was performed to adjust for potential baseline confounders including ASA status, frailty, surgical approach, and tumor stage. **Results**: Octogenarians exhibited a significantly higher prevalence of baseline frailty (23.2%, 32/138 evaluated vs. 5.8%, 35/605 evaluated, *p* < 0.001) and ASA physical status grade III–IV (70.3% vs. 45.7%, *p* < 0.001). Surgical approaches were comparable in both groups (*p* = 0.560). Overall, minimally invasive surgery rates (laparoscopic and robotic) reached 92.6% in octogenarians and 95.2% in younger controls. Specifically, robotic-assisted resection was implemented at equal rates between groups (71.1% vs. 71.1%). Overall 30-day postoperative complications were comparable (≥80: 42.0% vs. <80: 43.6%, *p* = 0.731), as were major complications (Clavien–Dindo > II: 9.4% vs. 11.1%, *p* = 0.572). Among patients undergoing primary intestinal anastomosis (*n* = 632), anastomotic leakage (3.5% vs. 3.1%, *p* = 0.835) and anastomotic bleeding (9.6% vs. 9.3%, *p* = 0.920) showed no significant differences. Multivariable logistic regression confirmed that advanced age ≥ 80 years was not an independent predictor of overall morbidity (aOR 0.92, 95% CI 0.60–1.41, *p* = 0.698). Median length of hospital stay (4 days, *p* = 0.391), 30-day readmissions (4.4% vs. 4.3%, *p* = 0.979), and 30-day in-hospital mortality (0.0% vs. 0.3%, *p* = 0.499) were equivalent. **Conclusions**: Elective curative-intent CRC resection in carefully selected octogenarians managed within a multidisciplinary pathway incorporating objective frailty assessment, prehabilitation, and minimally invasive techniques yields short-term postoperative outcomes, major complication rates, and hospital stays comparable to younger counterparts. Advanced chronological age alone should not preclude surgical resection in fit or mild-to-moderately frail older adults.

## Simple Summary

As the global population ages, an increasing number of older adults are diagnosed with colorectal cancer (CRC). However, elderly patients are frequently underrepresented in clinical trials, leading to a lack of clear evidence regarding the safety and effectiveness of standard treatments in this specific group. They are also associated with an increased incidence of major or medical complications. In the present study, we present our short-term surgical outcomes for patients undergoing CRC resection, showing no significant differences between octogenarians and younger counterparts. Systematic application of Enhanced Recovery After Surgery (ERAS) protocols, objective frailty assessment using the IF-VIG index, multimodal prehabilitation, and minimally invasive robotic surgery allow us to safely operate on carefully selected older patients with results comparable to younger controls.

## 1. Introduction

Colorectal cancer (CRC) remains the third most frequently diagnosed malignancy and the second leading cause of cancer-related mortality worldwide, representing a major public health challenge [1]. Driven by global demographic shifts, improved living conditions, and expanding life expectancy, the incidence of CRC is increasingly concentrated in older populations [2,3]. It is projected that by 2030, over 70% of all solid tumor diagnoses will occur in adults aged 65 years and older, with octogenarians (individuals aged 80 years and above) constituting the fastest-growing surgical demographic in modern surgical oncology practice [4,5].

Octogenarian patients represent a distinct, highly heterogeneous cohort characterized by physiological decline across multiple organ systems, diminished homeostatic reserve, and a high prevalence of geriatric syndromes, particularly physical frailty, cognitive impairment, and multimorbidity [6,7]. Historically, advanced chronological age has been viewed by clinicians and surgical teams as an independent predictor of adverse perioperative outcomes, high rates of severe postoperative morbidity, prolonged hospital stay, loss of functional independence, and elevated post-surgical mortality [8,9]. Consequently, ageism, therapeutic nihilism, undertreatment, or the inappropriate omission of radical curative-intent surgical resection have frequently been documented in real-world clinical practice [10,11]. Furthermore, older adults, especially those over 80 years, remain systematically underrepresented in randomized prospective clinical trials, severely limiting the availability of robust, high-level evidence to guide surgical decision-making and perioperative management in this vulnerable demographic [12].

Over the past two decades, modern surgical oncology and perioperative medicine have undergone a major paradigm shift [13]. The clinical management of surgical candidates has evolved from relying strictly on chronological age or simple physical status scales, such as the American Society of Anesthesiologists (ASA) score, toward comprehensive multidimensional evaluations [13,14]. The integration of formal Comprehensive Geriatric Assessment (CGA), validated frailty screening tools (such as the clinician-reported IF-VIG index), standardized Enhanced Recovery After Surgery (ERAS) pathways, and multimodal prehabilitation programs has fundamentally transformed perioperative care for older cancer patients.

Concurrently, surgical practice has experienced a rapid technological evolution with the widespread adoption of minimally invasive surgical techniques, most notably advanced robotic-assisted platforms [15,16,17]. Robotic surgery offers significant technical refinements over conventional laparoscopy, including high-definition three-dimensional binocular visualization, articulated endowrist instrumentation with seven degrees of freedom, ergonomic stability, and fine motion filtration [17]. These technological advantages facilitate meticulous mesorectal and complete mesocolic excision (CME) with enhanced preservation of autonomic pelvic nerves and crucial vascular structures, minimizing tissue trauma, reducing intraoperative blood loss, and attenuating the systemic inflammatory response [17,18]. Such physiological advantages are hypothesized to be particularly beneficial in frail, older patients who possess limited physiological reserves [19,20].

Despite these technological and perioperative advancements, controversy persists regarding whether radical colorectal resections in octogenarians carry an acceptable short-term safety profile compared to younger cohorts in real-world settings [21]. Many published series suffer from small sample sizes, heterogeneous inclusion criteria, failure to adjust for baseline frailty, or lack of standardized perioperative protocols [21,22].

Therefore, the primary objective of this retrospective comparative cohort study was to evaluate short-term surgical safety, 30-day postoperative morbidity, histopathological quality metrics, and health resource utilization between octogenarian (≥80 years) and younger (<80 years) patients undergoing elective curative-intent CRC resection within a high-volume, specialized colorectal surgery unit operating within a structured multidisciplinary pathway incorporating frailty screening, prehabilitation, ERAS, and robotic surgery [20,21,22,23,24,25].

## 2. Materials and Methods

### 2.1. Study Design and Patient Population

A single-center retrospective comparative cohort study was performed using a prospectively maintained institutional database at Hospital Universitari Parc Taulí (Sabadell, Barcelona, Spain), a tertiary university hospital catering to a catchment population of over 400,000 inhabitants. The study analyzed 742 consecutive adult patients who underwent elective surgical resection for primary colorectal adenocarcinoma between January 2021 and December 2025.

Ethics Statement: This study was conducted in accordance with the ethical principles originating in the Declaration of Helsinki. Ethical review and approval were waived for this study by the Institutional Review Board because the analysis relied exclusively on secondary data extracted from a prospectively maintained, non-interventional clinical registry of the Colorectal Surgery Unit. All data were fully anonymized prior to extraction and analysis, ensuring that individual patients could not be identified, in compliance with national and European data protection laws.

### 2.2. Variable Definitions and Endpoints

#### 2.2.1. Baseline and Demographic Parameters

Age, sex, American society of anesthesiologists (ASA) physical status classification, and baseline physical frailty status. Baseline frailty was systematically evaluated preoperatively using the “Índice Fragilidad-VIG” (IF-VIG) instrument, with frailty defined as an IF-VIG score ≥ 0.2 points [6,7].

Here, 30-day mortality was defined as all-cause mortality occurring within 30 days following the index surgical procedure, capturing both deaths during the initial hospital stay and post-discharge mortality identified through regional electronic health records.

##### Preoperative Frailty Evaluation (IF-VIG) and Geriatric Assessment

Routine preoperative evaluation included systematic frailty screening using the validated IF-VIG index (Índice Fragliidad-VIG), a clinician-reported multidimensional instrument administered during the preoperative consultation with the surgeon or dedicated geriatric assessment clinic.

Patients with an IF-VIG score ≥ 0.20 were identified as frail. Frail patients underwent a formal Comprehensive Geriatric Assessment (CGA) led by a geriatrician to determine frailty severity (mild, moderate, or severe).

Patients with mild-to-moderate frailty were cleared for surgery following targeted medical optimization.

In patients presenting with severe, irreversible frailty (IF-VIG > 0.50) or profound functional dependence, radical surgical resection was generally considered contraindicated by the Multidisciplinary Tumor Board, and non-surgical, best supportive, or palliative management strategies were recommended.

#### 2.2.2. Surgical and Technical Variables

Primary surgical procedure, surgical approach (robotic, laparoscopic, open, or conversion to open), type of anastomosis, anastomotic technique (intracorporeal vs. Extracorporeal), and protective stoma creation.

#### 2.2.3. Histopathological Characteristics

Pathological T, N, and M stages (AJCC 8th Edition), total lymph node count, metastatic lymph node count, and presence of lymphovascular or perineural invasion.

#### 2.2.4. Postoperative Outcomes and Complications

Overall 30-day postoperative morbidity, major complications (Clavien–Dindo grade > II), specific surgical and systemic complications (anastomotic leak, intra-abdominal abscess, anastomotic hemorrhage, acute kidney injury, respiratory failure, postoperative delirium, and paralytic ileus), length of hospital stay (los), 30-day re-intervention, 30-day readmission, and 30-day in-hospital mortality.

For anastomotic complications (leakage and bleeding), procedure-appropriate denominators were strictly applied, restricted to patients who actually received a primary bowel anastomosis.

### 2.3. Statistical Analysis

Given the samples size, normal variable distribution was assumed. Continuous clinical parameters such as age and postoperative stay are presented as median and interquartile range [IQR]. The other continuous parameters are presented as mean and standard deviation. Categorical and binary variables are expressed as absolute counts and percentages n (%).

Bivariate comparisons between the two age cohorts (<80 vs. ≥80 years) were conducted using t-student for continuous variables (or the Mann–Whitney U test for non-parametric continuous variables) and the Pearson Chi-square test (or Fisher’s exact test when expected cell frequencies were <5) for categorical and binary variables.

To adjust for potential baseline confounding arising from marked imbalances in ASA status, frailty, surgical approach, and tumor stage, multivariable logistic regression models were constructed for primary outcomes (overall morbidity and major complications). Adjusted Odds Ratios (aOR) with 95% confidence intervals (95% CI) were computed. Missing data were evaluated transparently and reported with specific available denominators (n/N).

All statistical tests were two-tailed, and a *p*-value < 0.05 was defined as statistically significant. Statistical analyses were performed using Stata software (version 16.0, StataCorp LLC, College Station, TX, USA).

### 2.4. Preoperative Management

All patients cleared for surgery were integrated into a standardized institutional Enhanced Recovery After Surgery (ERAS) pathway based on ERAS Society guidelines [14].

Preoperatively, candidates participated in a multimodal prehabilitation program delivered during outpatient visits by a specialized nurse practitioner. It was the institutional standard during the period described. The program incorporated:

1. **Physical Exercise:** Structured home-based aerobic walking recommendations (minimum 30 min daily) combined with respiratory muscle training (incentive spirometry);
2. **Nutritional Optimization**: Systematic screening for malnutrition using the MUST tool, high-protein oral nutritional supplements for malnourished candidates, and preoperative carbohydrate loading;
3. **Medical and Lifestyle Optimization**: Smoking cessation counseling, alcohol restriction, and preoperative correction of anemia (intravenous iron administration);
4. **Patient Education**: Structured verbal and written educational booklets explaining post-surgical recovery milestones, early ambulation targets, and pain management strategies.

Intraoperatively and postoperatively, standardized ERAS care protocols were strictly maintained, including restrictive fluid therapy, avoidance of routine nasogastric tubes and abdominal drains, active hypothermia prevention, early postoperative oral fluid intake, opioid-sparing multimodal analgesia, and forced early mobilization starting on postoperative day 0 [14,25].

## 3. Results

### 3.1. Baseline Patient Characteristics

As anticipated, octogenarian patients exhibited a significantly higher burden of physiological risk and comorbidity. ASA physical status grade III–IV was present in 70.3% (*n* = 97) of the ≥80 cohort compared to 45.7% (*n* = 276) in the <80 cohort (*p* < 0.001). Baseline frailty was four times higher in octogenarians (23.2% vs. 5.8%, *p* < 0.001). Distribution by sex was balanced across both groups (58.0% male in ≥80 vs. 57.9% male in <80, *p* = 0.979). Table 1.

### 3.2. Surgical Techniques and Histopathological Features

Surgical and pathological data are detailed in Table 2. Right hemicolectomy was more frequently performed in octogenarians (42.0% vs. 34.2%). Minimally invasive approaches were successfully deployed at equivalent rates in both age cohorts (92.6% in ≥80 vs. 95.2% in <80, *p* = 0.560). Robotic surgery represented the predominant approach in both groups (71.1% in ≥80 vs. 71.1% in <80). Open conversion rates were low and statistically indistinguishable (laparoscopic conversion: 2.2% vs. 1.9%; robotic conversion: 1.5% vs. 1.4%, *p* = 0.560). Table 2.

The mean lymph node harvest was 22 nodes in both groups (*p* = 0.733). No statistically significant differences were found regarding pathological T stage (*p* = 0.136), *n* stage (*p* = 0.902), M stage (*p* = 0.677), or rates of lymphovascular and perineural invasion.

### 3.3. Postoperative Outcomes and Specific Complications

Short-term postoperative morbidity, hospital resource utilization, and 30-day outcomes are outlined in Table 3.

Overall 30-day morbidity was equivalent between octogenarians and younger patients (42.0% vs. 43.6%, *p* = 0.731). Severe complications (Clavien–Dindo grade >II) occurred in 9.4% of octogenarians compared to 11.1% in younger controls (*p* = 0.572).

When evaluating surgical complications using appropriate denominators restricted to patients with a primary bowel anastomosis (*n* = 632), anastomotic leakage occurred in 3.5% (4/115) of octogenarians versus 3.1% (16/517) of younger patients (*p* = 0.835). Anastomotic bleeding occurred in 9.6% (11/115) versus 9.3% (48/517) (*p* = 0.920).

Median length of hospital stay was identical at 4.0 days in both groups (IQR: 3.0–8.0 in ≥80 vs. 3.0–7.0 in <80, *p* = 0.391). Thirty-day readmission rates (4.4% vs. 4.3%, *p* = 0.979), surgical re-interventions (5.1% vs. 3.5%, *p* = 0.373), and 30-day in-hospital mortality (0.0% vs. 0.3%, *p* = 0.499) were equally comparable.

### 3.4. Multivariable Logistic Regression and Subgroup Analysis by Frailty Status

Multivariable logistic regression was performed to evaluate whether advanced age (≥80 years) independently predicted overall morbidity or major complications after controlling for ASA score, frailty status, surgical approach, and T stage.

In the adjusted model, age ≥ 80 years was not an independent risk factor for overall morbidity (aOR 0.92, 95% CI 0.60–1.41, *p* = 0.698) or major complications (aOR 1.12, 95% CI 0.55–2.29, *p* = 0.759).

When outcomes are stratified strictly by baseline frailty status across the entire cohort (*n* = 621 evaluated), frail patients (IF-VIG ≥ 0.20) demonstrate significantly worse post-surgical trajectories compared to non-frail counterparts:

- **Overall Morbidity:** 55.7% (34/61) in Frail vs. 40.7% (228/560) in Non-frail (*p* = 0.024);
- **Major Complications:** 18.0% (11/61) in Frail vs. 9.6% (54/560) in Non-frail (*p* = 0.041);
- **Length of Stay:** Median 6.0 days [IQR 4.0–10.0] in Frail vs. 4.0 days [IQR 3.0–7.0] in Non-frail (*p* = 0.001).

## 4. Discussion

The management of colorectal cancer in octogenarian patients represents a major challenge for contemporary surgical oncology, driven by demographic shifts and the expanding population of older adults [1,2,3,4,5]. Historically, advanced chronological age has been viewed as a primary risk factor for postoperative adverse events, often leading to therapeutic hesitation or exclusion from radical surgical care [8,9,10,11]. However, the central finding of this comparative cohort study is that selected octogenarians undergoing elective curative-intent CRC resection within a specialized pathway achieve short-term surgical safety, major complication rates, and length of stay comparable to younger counterparts, despite harboring a significantly higher baseline burden of physical comorbidity (ASA III–IV) and physical frailty (IF-VIG ≥ 0.20).

In multivariable logistic regression analysis adjusting for baseline clinical covariates (Table 4), advanced chronological age (≥80 years) was not an independent predictor of overall morbidity (aOR 0.92, 95% CI 0.60–1.41, *p* = 0.698) or major complications (aOR 1.12, 95% CI 0.55–2.29, *p* = 0.759) per se. Due to collinearity between high ASA physical status, advanced age, and frailty, individual adjusted effects were attenuated in the multivariable model.

However, when evaluating outcomes strictly stratified by baseline frailty status across the cohort (Table 5), frail patients (IF-VIG ≥ 0.20) exhibited significantly higher rates of overall morbidity (55.7% vs. 40.7%, *p* = 0.024), major complications (18.0% vs. 9.6%, *p* = 0.041), and longer median hospital stay (6.0 vs. 4.0 days, *p* = 0.001) compared to non-frail controls, supporting the clinical role of frailty screening over chronological age alone.”

Our results strongly support the ongoing paradigm shift from chronological age to biological reserve and multidimensional frailty assessment [6,7,13]. Frailty represents a state of heightened vulnerability to stressors resulting from physiological decline across multiple organ systems [6]. In our series, objective frailty screening using the clinician-reported IF-VIG index allowed precise identification of vulnerable candidates [26].

A key strength of our clinical model is the structured perioperative pathway. Routine integration of multimodal prehabilitation (nurse-led physical exercise, respiratory training, nutritional support, and educational counseling) alongside standardized ERAS protocols provides an essential physiological buffer against perioperative stress. While prehabilitation was part of our institutional standard pathway rather than an independently tested variable, preoperative optimization likely contributed to stabilizing functional reserve, enabling octogenarians to achieve post-surgical recovery milestones identical to younger patients, as reflected by an equivalent median hospital stay of 4 days and low 30-day readmissions (4.4%).

Technological optimization through minimally invasive surgery also played a central role [15,17,18]. Over 92% of octogenarians underwent minimally invasive resection, with 71.1% treated via robotic-assisted surgery [17,18]. Robotic surgery provides superior 3D visualization, tremor filtration, and wrist articulation, enabling precise dissection in constrained anatomical spaces [17,22,24]. The attenuated surgical trauma and inflammatory response associated with robotic surgery likely contributed to the low overall rate of major complications (9.4%) and zero 30-day in-hospital mortality observed in our octogenarian cohort [18,22,23].

Oncology quality metrics were also preserved in the elderly group. The mean lymph node count was 20.0 nodes in both cohorts (*p* = 0.733), exceeding the International Union Against Cancer (UICC) minimum recommendation of 12 nodes [1]. This confirms that surgical radicality and lymphadenectomy standardizations were not compromised due to advanced patient age.

This study possesses notable strengths, including a large cohort of 743 consecutive patients treated in a standardized clinical setting, high adoption rates of robotic technology, and comprehensive collection of technical and pathological variables. However, limitations must be acknowledged. First, its retrospective single-center design introduces potential selection bias regarding patients deemed fit for elective surgery versus those managed palliatively. Second, long-term survival metrics (overall and disease-free survival) and patient-reported outcome measures (PROMs) such as postoperative quality of life were beyond the scope of this short-term surgical report and warrant future prospective investigation.

## 5. Conclusions

When managed within a specialized multidisciplinary pathway incorporating objective frailty assessment (IF-VIG), multimodal prehabilitation, ERAS protocols, and minimally invasive robotic surgery, carefully selected octogenarian patients undergoing elective curative-intent colorectal cancer resection achieve short-term surgical outcomes, major complication rates, and length of hospital stay comparable to younger counterparts. Advanced chronological age alone should not serve as an automatic barrier to radical surgery. Future prospective multicenter trials are needed to evaluate long-term oncological survival, functional preservation, and quality of life in older surgical oncology patients.

The main limitations include the retrospective single-center design and potential selection bias toward fitter octogenarians. Prospective multicenter trials are needed to evaluate long-term oncological survival, longitudinal functional trajectories, quality of life (QoL), and the impact of multimodal prehabilitation on preserving autonomy in octogenarian patients.

## Figures and Tables

**Table 1:** Baseline demographic and clinical characteristics of the study population (*n* = 743).

Characteristic	All Patients (*n* = 743)	<80 Years (*n* = 605)	≥80 Years (*n* = 138)	*p*-Value ^1^
**Age (years)**, Median [IQR]	72.5 [63.4–78.2]	68.7 [61.7–74.5]	83.2 [81.2–85.7]	**<0.001**
**Sex**, n (%)				0.979
Male	430 (57.9%)	350 (57.9%)	80 (58.0%)	
Female	313 (42.1%)	255 (42.1%)	58 (42.0%)	
**ASA Physical Status**, n (%)				**<0.001**
ASA I	27 (3.7%)	27 (4.5%)	0 (0.0%)	
ASA II	338 (45.8%)	299 (49.4%)	39 (28.3%)	
ASA III	330 (44.7%)	252 (41.7%)	**78 (56.5%)**	
ASA IV	43 (5.8%)	24 (4.0%)	**19 (13.8%)**	
**Frailty Status**, n (%)	67 (9.0%)	35 (5.8%)	32 (23.2%)	**<0.001**

^1^ Mann–Whitney U test for continuous variables; Chi-square test for categorical variables. Bold *p*-values indicate statistical significance (*p* < 0.05).

**Table 2:** Surgical approach and histopathological features stratified by age group.

Variable	<80 Years (*n* = 605)	≥80 Years (*n* = 138)	*p*-Value ^1^
**Surgical Procedure**, n (%)			0.253
Right Hemicolectomy	207 (34.2%)	58 (42.0%)	
Extended Right Hemicolectomy	31 (5.1%)	8 (5.8%)	
Left Hemicolectomy	61 (10.1%)	15 (10.9%)	
Sigmoidectomy	110 (18.2%)	24 (17.4%)	
High Anterior Resection	52 (8.6%)	12 (8.7%)	
Low Anterior Resection	58 (9.6%)	6 (4.3%)	
Abdominoperineal Resection (Miles)	49 (8.1%)	12 (8.7%)	
Subtotal/Total Colectomy	37 (6.1%)	3 (2.2%)	
**Surgical Approach**, n (%)			0.560
Overall minimally invasive surgery (Robotic + Laparoscopic Surgery)	553 (95.2%)	125 (92.6%)	
Robotic Surgery	413 (71.1%)	96 (71.1%)	
Laparoscopic Surgery	140 (24.1%)	29 (21.5%)	
Open Surgery	9 (1.6%)	5 (3.7%)	
Laparoscopic Conversion to Open	11 (1.9%)	3 (2.2%)	
Robotic Conversion to Open	8 (1.4%)	2 (1.5%)	
**Pathological Parameters**			
***Pathological T Stage, n (%)***			0.136
T0/Tis (In situ)	41 (8.6%)	5 (4.3%)	
T1	40 (8.4%)	10 (8.6%)	
T2	112 (23.6%)	19 (16.4%)	
T3	212 (44.7%)	65 (56.0%)	
T4 (4a/4b)	69 (14.6%)	17 (14.7%)	
***Pathological N Stage, n (%)***			0.902
N0	305 (64.5%)	79 (68.1%)	
N1 (1a/1b/1c)	96 (20.3%)	21 (18.1%)	
N2 (2a/2b)	60 (12.7%)	13 (11.2%)	
NX	12 (2.5%)	3 (2.6%)	
***Pathological M Stage, n (%)***			0.677
M0	355 (93.7%)	85 (95.5%)	
M1	21 (5.5%)	3 (3.4%)	
MX	3 (0.8%)	1 (1.1%)	
Harvested Lymph Nodes, Mean ± SD	22.3 ± 10.8	21.9 ± 9.7	0.733
Metastatic Lymph Nodes, Mean ± SD	1.4 ± 4.1	1.2 ± 3.5	0.801
Vascular Invasion, n (%)	96 (20.1%)	24 (20.3%)	0.951
Lymphatic Invasion, n (%)	99 (20.7%)	22 (18.6%)	0.617
Perineural Invasion, n (%)	106 (22.1%)	22 (18.6%)	0.409

^1^ Chi-square test or Fisher’s exact test for categorical variables; Mann–Whitney U test for continuous variables.

**Table 3:** Postoperative 30-day outcomes and specific complications.

Postoperative Outcome	<80 Years (*n* = 605)	≥80 Years (*n* = 138)	*p*-Value ^1^
**Overall Morbidity**, n (%)	264 (43.6%)	58 (42.0%)	0.731
**Major Complications (CD > II)**, n (%)	67 (11.1%)	13 (9.4%)	0.572
**Specific Complications**, n (%)			
Anastomotic Leakage ^2^	16 (3.1%)	4 (3.5%)	0.835
Anastomotic Bleeding ^2^	48 (9.3%)	11 (9.6%)	0.920
Intra-abdominal Abscess	20 (3.3%)	2 (1.5%)	0.246
Paralytic Ileus	27 (4.5%)	5 (3.6%)	0.661
Acute Kidney Injury	7 (1.2%)	2 (1.5%)	0.777
Respiratory Failure	2 (0.3%)	1 (0.7%)	0.510
Postoperative Delirium	2 (0.3%)	1 (0.7%)	0.510
**Resource Utilization and Safety Metrics**			
Length of Hospital Stay (days), Median [IQR]	4.0 [3.0–7.0]	4.0 [3.0–8.0]	0.391
30-day Readmission, n (%)	26 (4.3%)	6 (4.4%)	0.979
30-day Surgical Reintervention, n (%)	21 (3.5%)	7 (5.1%)	0.373
30-day In-hospital Mortality, n (%)	2 (0.3%)	0 (0.0%)	0.499

^1^ Chi-square test or Fisher’s exact test for binary outcomes; Mann–Whitney U test for length of stay. ^2^ Anastomotic leakage and anastomotic bleeding denominators are restricted to patients with a primary bowel anastomosis (*n* = 632). 115 of octogenarians versus 517 in younger patients.

**Table 4:** Multivariable logistic regression analysis for postoperative morbidity endpoints.

Outcome	Adjusted Odds Ratio (aOR)	95% Confidence Interval	*p*-Value ^1^
**Overall 30-Day Morbidity**			
Age ≥ 80 years (vs. <80 years)	0.92	0.60–1.41	0.698
ASA Physical Status III–IV (vs. I–II)	1.00	0.72–1.39	0.996
Frailty Status (IF-VIG ≥ 0.20 vs. < 0.20)	0.79	0.46–1.38	0.411
Surgical Approach (Minimally Invasive vs. Open)	0.71	0.32–1.58	0.402
Pathological Tumor Stage (T3–T4 vs. T1–T2)	1.18	0.85–1.64	0.324
**Major complications (Clavien–Dindo Grade > II)**			
Age ≥ 80 years (vs. <80 years)	1.12	0.55–2.29	0.759
ASA Physical Status III–IV (vs. I–II)	1.10	0.64–1.90	0.735
Frailty Status (IF-VIG ≥ 0.20 vs. <0.20)	0.24	0.06–1.05	0.058
Surgical Approach (Minimally Invasive vs. Open)	0.54	0.17–1.72	0.298
Pathological Tumor Stage (T3–T4 vs. T1–T2)	1.35	0.78–2.34	0.285

^1^ Multivariable logistic regression model calculated on complete cases with available baseline IF-VIG frailty assessment, ASA classification, surgical approach, pathological stage and postoperative complication data.

**Table 5:** Postoperative outcomes stratified strictly by baseline frailty status.

Postoperative Outcome	Non-Frail (IF-VIG < 0.20) (*n* = 560)	Frail (IF-VIG ≥ 0.20) (*n* = 61)	*p*-Value ^1^
Overall 30-Day Morbidity, n (%)	228 (40.7%)	34 (55.7%)	**0.024**
Major Complications (Clavien–Dindo Grade > II), n (%)	54 (9.6%)	11 (18.0%)	**0.041**
Length of Hospital Stay (days), Median [IQR]	4.0 [3.0–7.0]	6.0 [4.0–10.0]	**0.001**
30-Day Unplanned Readmission, n (%)	23 (4.1%)	5 (8.2%)	0.154

^1^ Chi-square test or Fisher’s exact test for binary outcomes; Mann–Whitney U test for length of stay. Bold *p*-values indicate statistical significance (*p* < 0.05).

## Data Availability

The raw dataset of this study is not available due to the privacy of the patients and lack of informed consent.
